# Analysis of risk factors and preventive strategies for intracranial infection after neuroendoscopic transnasal pituitary adenoma resection

**DOI:** 10.1186/s12868-021-00688-3

**Published:** 2022-01-03

**Authors:** Xin Huang, Xuejun Zhang, Jian Zhou, Gang Li, Gang Zheng, Lei Peng, Ziwei Yan, Shaojun Chen

**Affiliations:** 1 Department of Neurosurgery, The People’s Hospital of China Three Gorges University, Yichang, 443000 China; 2 Department of Neurosurgery, Dangyang People’s Hospital, Yichang, China; 3 Department of Ultrasound Diagnostics, The People’s Hospital of China Three Gorges University, Yichang, China

**Keywords:** Pituitary adenoma, Neuroendoscope, Intracranial infection, Transnasal approach, Risk factors

## Abstract

**Objective:**

To analyse the risk factors for intracranial infection after neuroendoscopic transnasal pituitary adenoma resection (NTPAR) to provide a reference for the prevention and treatment of postoperative intracranial infection.

**Methods:**

The clinical data of 387 patients who underwent NTPAR in the Department of Neurosurgery of the First People’s Hospital of Yichang from March 2013 to March 2021 were retrospectively analysed. The patients were divided into an infected group and a noninfected group according to the occurrence of intracranial infection. The detailed clinical data of the two groups were collected. Univariate and multivariate logistic regression was used to analyse the risk factors for intracranial infection after NTPAR.

**Results:**

Among the 387 surgical patients, 32 patients (8.27%) were in the intracranially infected group and 355 patients (91.73%) were in the noninfected group. The results of the univariate analysis suggested that age > 45 years, tumour size > 1 cm, operation time > 240 min, blood loss > 400 ml, Kelly Grade of cerebrospinal fluid (CSF) leakage > Grade 2, postoperative CSF leakage, lumbar cistern drainage and blood transfusion were the influencing factors for postoperative intracranial infection, while the results of multivariate logistic regression analysis implied that intraoperative CSF leakage (Kelly Grade > 2) and postoperative CSF leakage were independent influencing factors for intracranial infection after NTPAR, and perioperative use of antibiotics was an independent protective factor for postoperative intracranial infection.

**Conclusions:**

There are a variety of risk factors for intracranial infection after NTPAR, which indicates that it is necessary to develop different repair strategies for CSF leakage according to the Kelly Grade, timely treatment of postoperative CSF leakage and perioperative use of antibiotics. These measures have been shown to effectively reduce the probability of intracranial infection after NTPAR.

## Introduction

Pituitary adenomas are common benign tumours of the nervous system, second only to gliomas and meningiomas, accounting for approximately 15% of intracranial tumours [1]. With the exception of prolactinomas, pituitary adenomas usually require surgical treatment [2]. With the development of high-definition and 3D endoscopic technology, neuroendoscopic transnasal pituitary adenoma resection (NTPAR) has become the standard surgical method [3]. Although endoscopic transnasal surgery has the advantages of less trauma, a clear field of vision and a low incidence of complications, some patients still have complications, such as postoperative cerebrospinal fluid (CSF) leakage, intracranial infection, intracranial haemorrhage, permanent diabetes insipidus, and optic nerve injury [4]. In the transnasal approach, there are usually a variety of bacteria colonizing the nasal sinus cavity. Bacteria can easily enter the brain through surgical instruments or CSF to cause intracranial infection. Therefore, the transnasal approach has a high potential risk of intracranial infection, with an incidence of 3.59% [5]. Intracranial infection can aggravate a patient’s condition and is even life-threatening in severe cases, with a mortality rate as high as 3–33% [6]. Even if intracranial infection is completely cured, patients may be left with varying degrees of neurological dysfunction, and intracranial infection increases medical risks and medical costs [7]. However, there are few studies on the risk factors for and prevention of intracranial infection after NTPAR either at home or abroad. This study was a retrospectively analysis of the clinical data of 387 inpatients with NTPAR in the Department of Neurosurgery of our hospital from March 2013 to March 2021. The related risk factors for intracranial infection after NTPAR were analysed, and the independent risk factors for intracranial infection were determined to provide relevant intracranial infection prevention strategies.

## Data and methods

### Research patients

The clinical data of 387 NTPAR patients in the Department of Neurosurgery of the First People’s Hospital of Yichang City from March 2013 to March 2021 were collected retrospectively. According to the occurrence of postoperative intracranial infection, the patients were divided into an infected group (n = 32) and a noninfected group (n = 355). This study is in line with the relevant ethical standards and was approved by the hospital ethics committee.

Inclusion criteria: (1) NTPAR operation was performed for the first time; (2) the Diagnostic Standards for Hospital Infection (Trial) criteria for central nervous system infection, established by the Ministry of Health of the People’s Republic of China in 2001, were met [8]; (3) no intracranial infection occurred before the operation; (4) complete clinical data; and (5) a diagnosis of pituitary adenoma at an age older than 18 years. The exclusion criteria were as follows: (1) patients with a postoperative pathological diagnosis of nonpituitary adenoma; (2) patients with a history of central nervous system infection, such as meningitis, ventriculitis, intracranial abscess, and intraspinal infection, before surgery; and (3) incomplete clinical data. The diagnostic criteria for postoperative intracranial infection are presented in Table 1 [8]. In patients with suspected potential infection, auxiliary examination should be combined with a comprehensive evaluation of clinical symptoms.

**Table 1:** Diagnostic criteria for intracranial infection

Definitive diagnosis of an infection	Aetiological diagnosis: culture of pathogenic bacteria in CSF or positive immunological identification of pathogenic microorganisms in CSF
Diagnosis of a potential infection	(1) Fever (temperature ≥ 38 °C), headache, stiff neck, meningeal signs, cranial nerve signs, and/or irritability with no other recognized cause (2) Examination of CSF released by lumbar puncture: cloudy, intracranial pressure > 200 mmH_2_O, white blood cell count > 50 × 10^6^/L, glucose < 2.2 mmol/L or a CSF glucose/serum glucose ratio < 0.4, CSF protein > 0.45 g/L [9] (3) CT or MRI findings: meningitis often indicates diffuse brain oedema; ventriculitis indicates ventricular dilatation or fluid level in the ventricle; circular enhancement can be seen in brain abscesses

CT: computed tomography; MRI: magnetic resonance imaging

## Methods

Data from patients who met the inclusion criteria were retrospectively collected through the hospital electronic medical record system. The data collected included sex, age, body mass index (BMI), a history of hypertension, a history of diabetes, American Society of Anesthesiologists (ASA) score, tumour size, operation time, blood loss, CSF leakage during operation (Kelly Grade, see Table 2), postoperative CSF leakage, sinusitis, perioperative use of antibiotics, lumbar cistern drainage and blood transfusion.

**Table 2:** Kelly criteria for CSF rhinorrhoea

CSF leakage classification	Description
Grade 0	No CSF rhinorrhoea, confirmed by Valsalva manoeuvre
Grade 1	CSF rhinorrhoea as small as teardrops, confirmed by Valsalva manoeuvre
Grade 2	Moderate CSF rhinorrhoea with obvious saddle septum defect
Grade 3	Major CSF rhinorrhoea, large saddle septal defect

### Determination of the univariate analysis variable entry points

As shown in Table 3, according to the data of this study and those of related literature, age = 45 years, BMI = 24.9, ASA score > 2, tumour size = 1 cm, operation time = 240 min, and blood loss = 400 ml were selected as entry points for the univariate analysis.

### Statistical analysis

SPSS 20.0 software was used for statistical analysis. The counted data are expressed as the percentage or number of cases, and between-group comparisons were done performed with the X^2^ test. Normally distributed measurement data are expressed as $\bar{x}$  ± s, and between-group comparisons were performed with a t-test. One-way analysis of variance (ANOVA) was used to analyse the risk factors for postoperative intracranial infection, and the variables with significant differences were selected and included in multivariate logistic regression analysis. Differences with P < 0.05 were considered statistically significant (P < 0.05).

## Results

### Between-group comparisons of general clinical data

A total of 387 patients with pituitary adenomas were enrolled in this study, all of whom underwent NTPAR. The clinical manifestations included headache (n = 183), decreased vision (n = 98), amenorrhea syndrome (n = 33), and acromegaly (n = 19); 54 patients were asymptomatic. Pituitary dynamic contrast-enhanced magnetic resonance imaging (MRI) was performed before the operation, and tumour measurements, three-dimensional computed tomography (CT) of the head and computed tomography angiography (CTA) examination of the head were performed. No significant differences in sex, age, BMI, hypertension history, diabetes history, ASA score or sinusitis were noted between the two groups (P > 0.05, Table 3).

**Table 3:** Clinical data of 387 patients with pituitary tumours

Items	Noninfected group (n = 355)	Infected group (n = 32)	P (noninfected group vs. infected group)
Sex (male, %)	47.89%	37.50%	0.274^a^
Age (x ± s, years)	44.84 ± 13.28	48.81 v 14.81	0.151^b^
BMI (x ± s, kg/m^2^)	22.16 ± 2.61	22.26 ± 2.27	0.815^b^
History of hypertension (%)	34.37%	24.51%	0.219^a^
History of diabetes (%)	15.78%	25%	0.179^a^
ASA score (x ± s)	1.88 ± 0.88	1.93 ± 0.76	0.697^b^
Tumour size (x ± s, cm)	2.02 ± 0.99	2.43 ± 1.02	0.035^b^
Small adenoma (case)	70	2	
Large adenoma (case)	270	26	
Giant adenoma (case)	15	4	
Operation time (x ± s, minutes)	198.51 ± 81.68	245.5 ± 86.57	0.005^b^
Bleeding volume (x ± s, ml)	267.79 ± 111.55	397.81 ± 141.96	0.000^b^
Kelly Grade of intraoperative CSF leakage (x ± s, Grade)	0.96 ± 0.84	1.50 ± 1.05	0.007^b^
0 (cases)	109	6	
1 (case)	175	11	
2 (cases)	48	8	
3 (cases)	23	7	
Postoperative CSF leakage (cases, %)	10.42%	28.12%	0.001^a^
Sinusitis (cases, %)	16.34%	31.25%	0.091^a^
Use of antibacterial drugs during perioperative period (%)	92.11%	81.25%	0.038^a^
Drainage of lumbar cistern (%)	5.07%	15.62%	0.016^a^
Blood transfusion (%)	5.35%	15.62%	0.021^a^

^a^ is the X^2^ value and ^b^ is the Z value

### Intracranial infection rate and pathogen distribution after NTPAR

Among the 387 patients with NTPAR, 32 patients developed intracranial infection, and the infection rate was 8.27%. Among them, 30 patients had meningitis, and 2 patients had ventriculitis. Of the 32 patients with infection, 5 patients were positive in CSF culture, with a positive rate of 15.6%, including 2 cases of *Acinetobacter baumannii* infection, 1 case of *Staphylococcus epidermidis* infection, 1 case of *Staphylococcus aureus* infection and 1 case of *Pseudomonas aeruginosa* infection.

### Analysis of related factors affecting patients with intracranial infection after NTPAR

The patients were divided into an infected group and a noninfected group according to the occurrence of intracranial infection after the operation. The effects of sex, age, BMI, a history of hypertension, a history of diabetes, tumour size, ASA score, operation time, blood loss, Kelly Grade of CSF rhinorrhoea, postoperative CSF leakage, sinusitis, preoperative use of antibiotics, lumbar cistern drainage and blood transfusion on postoperative intracranial infection were compared by single-factor analysis of variance. Univariate analysis indicated that age > 45 years, tumour size > 1 cm, operation time > 240 min, blood loss > 400 ml, Kelly Grade of CSF leakage > Grade 2, postoperative CSF leakage, lumbar cistern drainage and blood transfusion were the influencing factors for postoperative intracranial infection. Perioperative use of antibiotics was a protective factor against postoperative intracranial infection (see Table 4).

**Table 4:** Univariate analysis of patients with intracranial infection after NTPAR

	Influencing factors	Infected group (n = 32)	Noninfected group (n = 355)	Infection rate (%)	X^2^ value	P value
Sex	Male	12	170	6.59	1.271	0.274
	Female	20	185	9.76		
Age (years)	> 45	19	140	11.95	4.821	0.028
	≤ 45	13	215	5.70		
BMI (kg/m^2^)	> 24.9	8	102	7.27	0.201	0.654
	≤ 24.9	24	253	8.66		
History of hypertension	Yes	11	87	11.11	1.512	0.219
	No	21	268	7.23		
History of diabetes	Yes	8	56	12.5	1.810	0.179
	No	24	299	7.43		
ASA score	> 2	8	117	6.40	0.794	0.737
	≤ 2	24	238	9.16		
Tumour size (cm)	> 1	29	266	9.83	3.990	0.046
	≤ 1	3	89	3.26		
Operation time (min)	> 240	16	85	15.84	10.333	0.001
	≤ 240	16	270	5.59		
Bleeding volume (ml)	> 400	9	39	18.75	7.936	0.005
	≤ 400	23	316	6.78		
Kelly Grade	> 2	6	22	21.43	6.890	0.009
	≤ 2	26	333	7.24		
Postoperative CSF leakage	Yes	10	37	21.27	11.934	0.001
	No	22	318	6.47		
Sinusitis	Yes	9	58	13.43	2.849	0.091
	No	23	297	7.19		
Antibacterial drug use during perioperative period	Yes	26	327	7.37	4.322	0.038
	No	6	28	17.65		
Lumbar cistern drainage	Yes	5	18	21.74	5.85	0.016
	No	27	337	7.42		
Blood transfusion	Yes	5	19	20.83	5.325	0.021
	No	27	336	7.44		

### Multivariate logistic regression analysis of patients with intracranial infection after NTPAR

The findings from the multivariate logistic regression analysis suggested that intraoperative CSF leakage (Kelly Grade > 2) and postoperative CSF leakage were independent influencing factors for intracranial infection after NTPAR. Perioperative use of antibiotics was an independent protective factor for intracranial infection after NTPAR (see Table 5).

**Table 5:** Multivariate logistic regression analysis of patients with intracranial infection after NTPAR

Relevant factors	Β value	SE	Wald	OR	P value	95% CI
Kelly Grade of intraoperative CSF leakage > 2	2.518	1.164	4.677	12.405	0.031	1.266~121.534
Postoperative CSF leakage	1.130	0.568	3.950	3.094	0.047	1.016~9.427
Perioperative use of antibacterial drugs	-1.147	0.555	4.260	0.318	0.039	0.107~0.944

SE: standard error; OR: odds ratio; CI: confidence interval

## Discussion

Intracranial infection not only is a common complication after NTPAR but also remains a critical problem for neurosurgeons [10]. Intracranial infection can aggravate a patient’s condition, be life-threatening, and prolong a patient’s length of hospital stay. Even if intracranial infection is completely cured, patients may be left with varying degrees of neurological dysfunction, and intracranial infection increases medical risks and medical costs. Therefore, how to prevent and reduce the incidence of intracranial infection has important clinical significance. In recent years, there has been some controversy in clinical research on the risk factors for intracranial infection after NTPAR. Guo K et al. showed that the intracranial infection rate after NTPAR was 3.59%. Postoperative CSF leakage was the influencing factor of intracranial infection [5]. Xu et al. confirmed that the intracranial infection rate after NTPAR was 4.86%, and risk factors included bleeding > 120 ml, CSF leakage, diabetes, preoperative use of hormones, operation time, and macroadenoma [11]. In this study, 32 patients developed postoperative intracranial infection, and the infection rate was 8.27%, which was higher than that reported in the literature, which may be related to the patients’ conditions and other potential diseases. Additionally, this work also reports that the degrees of intraoperative CSF leakage and postoperative CSF leakage were also risk factors for intracranial infection after NTPAR and proposes specific prevention and treatment measures according to the risk factors for intracranial infection. Perioperative use of antibiotics was an independent protective factor for postoperative intracranial infection.

### Univariate analysis of intracranial infection after NTPAR

The results of the univariate analysis implied that intracranial infection is caused by the following factors: (1) Macroadenoma and tumour size > 1 cm were risk factors for intracranial infection [12]. Macroadenomas and giant adenomas often dilate to the third ventricle. During tumour resection, it is easy to cause an opening in the sellar septum and the floor of the third ventricle, resulting in high-flow CSF leakage. In the surgical removal of macroadenomas and giant adenomas, the first is to remove them along the edge of the sellar septum to prevent it from collapsing prematurely and avoid damaging the arachnoid of the suprasellar cistern as much as possible, which is helpful to reduce CSF leakage. The treatment of CSF leakage will be described in detail below. (2) An operation time of more than 4 h, indicating that the prolonged exposure time at the site of the surgical incision and the dehydration of incision tissue, increased the risk of bacterial contamination. Cheng H noted that as the operation time increased, the probability of intracranial infection following neurosurgery increased by 24%, and with operation times of more than 3 to 4 h, the probability of intracranial infection doubled [13]. Detailed preoperative planning, good teamwork and surgeon skill improvement are all helpful to shorten the operation time. (3) Blood loss ≥ 400 ml and blood transfusion were risk factors for intracranial infection. The infection probability of perioperative allogeneic blood transfusion is 7 to 10 times higher than that of no blood transfusion or autologous blood transfusion. Long MY claimed that autologous blood transfusion can increase the levels of TNF-α and complement C3 in patients, thereby enhancing their immunity against infection [14]. Preoperative autologous blood storage for patients and intraoperative autologous blood recovery for patients with a large amount of bleeding are performed. In addition, the amount of intraoperative bleeding should be restricted. (4) Furthermore, other risk factors for intracranial infection have been discussed, such as age, BMI, ASA score, and sinusitis, but there is no consensus on whether these are risk factors for postoperative intracranial infection. In this study, the patients ultimately included in the study were relatively older, and patients older than 45 years old are more prone to intracranial infection, as reported by Fang et al. [15], but Wang et al. found that patients younger than 45 years old were more likely to develop intracranial infection [16]. There was no significant difference in the incidence of intracranial infection among patients with a BMI ≥ 24.9 kg/m^2^ in the univariate analysis, but it was considered to be a risk factor for intracranial infection [17]. Therefore, it is considered that for patients with a BMI ≥ 24.9 kg/m^2^ and other risk factors for intracranial infection, it is necessary to prolong the perioperative antibiotic use time. An ASA score > 2 was not a risk factor for intracranial infection, which is consistent with the study of Wang et al.  [16]. Kingsley et al. [18] stated that an ASA score > 2 was a risk factor for intracranial infection in patients with diabetes. Based on the relevant literature studies in patients with other basic diseases, it is suggested that perioperative control of reoperation is beneficial to reduce the risk of postoperative intracranial infection in patients with an ASA score > 2. Acute and chronic sinusitis can lead to intracranial abscesses, meningitis and other intracranial complications [19], and NTPAR is not recommended in the treatment of pituitary adenomas during acute sinusitis. The nasal cavities of most patients with chronic sinusitis are colonized with bacteria, with the most common being *Staphylococcus aureus*. Iodophor disinfection of the nasal cavity can effectively remove most of the bacteria in the nasal cavity, and the probability of intracranial infection after NTPAR is very low [20].

### Multivariate logistic regression analysis of the influence of intracranial infection

Intraoperative CSF leakage (Kelly Grade > 2) and postoperative CSF leakage were independent influencing factors for intracranial infection after NTPAR, and perioperative use of antibiotics was a protective factor against postoperative intracranial infection. During transnasal pituitary adenoma resection, the rate of intraoperative CSF leakage can reach 17.9%, and the rate of postoperative CSF leakage is 2.7% [21]. As in this study, there was a significant correlation between intraoperative and postoperative CSF leakage and postoperative intracranial infection [17]. According to the Kelly classification, intraoperative CSF leakage is divided into Grades 0–3, and different repair strategies are performed for each grade [22]: (1) Grade 0 CSF leakage is repaired by filling the tumour cavity with absorbable haemostatic gauze, supporting the tumour cavity by an expanded gelatine sponge, restoring the nasal mucosa, and supporting the nasal cavity on the operating side with iodoform gauze. (2) The “extrusion method” is recommended for the repair of Grade 1 CSF leakage. First, the absorbable haemostatic gauze and a large piece of gelatine sponge are extruded at the leak; artificial dura mater is applied to the bottom of the saddle, which can closely fill the tumour cavity; and the nasal mucosa is restored. Iodoform gauze is used to support the nasal cavity on the operating side. (3) Grade 2 CSF leakage is repaired with “sandwiches” to fill the tumour cavity with haemostatic gauze, a large gelatine sponge and autologous fat. In the middle layer, the artificial dura mater is fixed with biological glue, the mucosa of the sphenoid sinus is removed, and then autologous fat is used to fill the tumour cavity and sphenoid sinus cavity to avoid dead space. The outer layer is a biological glue-fixed gelatine sponge to support autologous fat. The nasal mucosa is restored. Iodoform gauze is used to support the operating side of the nasal cavity and is generally recommended to remain in place for more than 5 days. (4) Grade 3 CSF leakage requires “skull base reconstruction”. First, the medial side of the dura leakage of the skull base is filled with autologous fat. The gelatine sponge should cover more than the fat to the edge of the bone defect and cover the artificial dura mater. Subsequently, autogenous fascia packing or bony support is used to repair the skull base bone defect, and the nasal septum pedicled mucosal flap completely covers the sphenoid sinus. Then, autologous fat is used to cover the mucous flap again, a gelatine sponge is fixed to support the fat, and both nasal cavities are filled with iodophor gauze. It is worth noting that dead space should be avoided, and gauze is recommended to remain in place for more than 2 weeks. (5) Postoperative CSF leakage was an independent risk factor for intracranial infection. The bacteria in the nasal sphenoid sinus easily enter the brain in a retrograde manner through the leak, and patients with postoperative CSF leakage are prone to intracranial infection recurrence [23]. If CSF leakage has been found during the operation, it should be repaired in a timely manner. Skull base reconstruction is also recommended for severe collapse of the sellar septum with no CSF leakage during the operation. Some patients have no CSF leakage during the operation; instead, CSF leakage occurs after the operation, which may be related to postoperative forced defecation, sneezing, coughing, etc. Postoperative CSF leakage should be actively treated with absolute bed rest, prophylactic use of antibiotics and lumbar cistern drainage if necessary. Some scholars suggest that prophylactic lumbar cistern drainage of CSF during the perioperative period can reduce the incidence of CSF leakage [24]. Our experience at our centre is that lumbar cistern drainage itself is also a risk factor for intracranial infection, but for patients with CSF leakage, lumbar cistern drainage can reduce the necessity for secondary reconstruction surgery. Timely repair of CSF leakage is performed in patients with no improvement in CSF leakage after conservative treatment for 2 weeks. (6) Perioperative use of antibiotics was an independent protective factor for postoperative intracranial infection. Prophylactic use of antibiotics can reduce the incidence of postoperative intracranial infection from 8.80 to 1.90% [25]. Appropriate antibiotics should be selected according to the results of bacterial CSF culture, but the bacterial culture of CSF of most patients with intracranial infection is negative [26]. In most cases, drugs are applied based on clinical experience, and antibiotics have certain side effects and even increase the drug resistance of pathogenic bacteria. Therefore, the use of antibiotics must be considered comprehensively, drugs with narrow antibacterial spectra and high specificity should be selected as much as possible, and it is best to avoid combined drug use. Perioperative use of single antibiotics is sufficient to prevent intracranial infection [27], and vancomycin should be the first choice for patients with suspected intracranial infection after operation [28].

## Limitations of the study

This study has limitations. This study was a retrospective analysis of the data from patients from a single centre, but some of our findings have been confirmed in multicentre studies. In addition, the follow-up period of this study was short, with no long-term follow-up of patients; therefore, the long-term effect of intracranial infection on patients undergoing NTPAR is not clear.

## Conclusions

There are a variety of risk factors for intracranial infection after NTPAR. Age > 45 years, tumour size > 1 cm, operation time > 240 min, blood loss > 400 ml, Kelly Grade of CSF leakage > Grade 2, postoperative CSF leakage, lumbar cistern drainage and blood transfusion were the influencing factors for postoperative intracranial infection. Perioperative use of antibiotics was a protective factor against postoperative intracranial infection. Intraoperative CSF leakage (Kelly Grade > 2) and postoperative CSF leakage were independent influencing factors for intracranial infection after NTPAR. Perioperative use of antibiotics was an independent protective factor for postoperative intracranial infection. Finally, the analysis of the causes of different risk factors can be helpful for clinicians to formulate individualized and accurate prevention strategies, which can effectively reduce the probability of intracranial infection after NTPAR.

## Data Availability

The datasets used and/or analysed during the current study are available from the corresponding author on reasonable request.
